# Effects of the Prevention Program “HateLess. Together against Hatred” on Adolescents’ Empathy, Self-efficacy, and Countering Hate Speech

**DOI:** 10.1007/s10964-023-01753-2

**Published:** 2023-02-25

**Authors:** Sebastian Wachs, Norman Krause, Michelle F. Wright, Manuel Gámez-Guadix

**Affiliations:** 1 Department of Educational Sciences, University of Potsdam, Potsdam, Germany; 2 National Anti-Bullying Research and Resource Centre, Dublin City University, Dublin, Ireland; 3 Department of Psychology, DePaul University, Chicago, IL USA; 4 Department of Biological and Health Psychology, Autonomous University of Madrid, Madrid, Spain

**Keywords:** Hate speech, Counter-speech, Prevention, Empathy, Self-efficacy, Diversity

## Abstract

Currently, there is a lack of empirically evaluated prevention programs targeting hate speech among adolescents. This is problematic because hate speech jeopardizes adolescents’ well-being and social integration. To this end, this study aims to evaluate the short-term effects of the newly developed anti-hate speech prevention program, “HateLess. Together against Hatred”, on adolescents’ empathy, self-efficacy, and counter-speech. Eight hundred and twenty adolescents between 12 and 16 (*M* = 13.27, *SD* = 1.04) from 11 German schools participated in this study. More specifically, 567 adolescents participated in the one-week prevention program, and 253 participants were assigned to the control group. Repeated measures ANOVAs showed that HateLess was successful, as there was a significant increase in empathy, self-efficacy, and counter-speech in the intervention group from the pretest (T1) to the posttest (T2) one month after the intervention. In contrast, no changes were found among adolescents in the control group. A multilevel mediation model revealed that the effect of being a member of the intervention group on counter-speech was partially mediated via empathy and self-efficacy. The findings indicate that HateLess is an effective, cost-efficient approach to enhance adolescents’ counter-speech directly and indirectly by altering the skills they need to become informed citizens in democratic societies.

## Introduction

Hate speech among youth is a serious and widespread problem that plagues schools worldwide (Kansok-Dusche et al., 2022). This is problematic because being the target of hate speech is related to lower well-being and poses threats to the personal development of adolescents (Krause et al., 2021; Wachs, Gámez-Guadix et al., 2022). In addition, hate speech exposure might lead to political radicalization and increase prejudices toward minorities (Soral et al., (2018)). Schools have a dual role regarding hate speech. On one hand, schools expose students to diversity, thus increasing risks for social conflicts. On the other hand, schools are predestined to prevent hate speech through the possibility of democratic education, opportunities to learn social skills, and tolerance. In German schools, however, teachers are often overwhelmed in dealing with this relatively new phenomenon (Krause et al., in press). At the same time, there is a lack of theory-based and empirically evaluated programs targeting hate speech among adolescents (Seemann-Herz et al., 2022). To this end, the present study investigates the short-term effects of a newly developed anti-hate speech prevention program on adolescents’ empathy toward victims of hate speech, their self-efficacy in dealing with hate speech incidents, and their engagement in counter-speech. In addition, the present study aims to understand how participation in the prevention program alters adolescents’ counter-speech by investigating the mediating role of empathy and self-efficacy.

### Background, Aims, and Development of the Prevention Program “HateLess”

The term hate speech refers to any harmful communicative form of expression (e.g., words, graffiti, comments on social media, images) that deliberately promote, justifies, or disseminates hatred or prejudices toward particular social groups and minorities (e.g., LGBTQI+people, people of color) in the online or offline world (Kansok-Dusche et al., 2022). According to a recent study in Germany, 58% of the participants reported that they had witnessed hate speech in schools at least once within 12 months, 27% reported that they were the target of hate speech, and 19% stated that they had perpetrated hate speech themselves (Castellanos et al., in press).

Hate speech exhibits both conceptual and empirical overlaps with bullying. Bullying is often described as a repeated, harmful behavior against targets that cannot easily defend themselves occurring in relatively stable social groups (e.g., within schools or classes) and resulting from interpersonal conflicts between members of those social groups (Olweus, 1978). Unlike bullying, hate speech can be carried out as a single act among people who do not know each other and are not members of one social group. In addition, hate speech is related to prejudicial attitudes and group-focused enmity as it targets people based on actual or assigned group characteristics and is often triggered by social or political events (Kansok-Dusche et al., 2022; Piatkowska & Stults, 2022). Although previous research found an overlap between hate speech and bullying (Ballaschk et al., 2022; Wachs et al., 2019), evidence supports the assumption that both phenomena present specific distinguishable constructs (Bedrosova et al., 2022). These findings highlight the need for prevention programs explicitly targeting hate speech in schools.

The prevention program “HateLess. Together against hatred” (the term HateLess is used for brevity) is designed for adolescents in Grades 7–9. The overarching goal of HateLess is to prevent hate speech perpetration and victimization among adolescents and equip adolescents with the needed skills to stand up against hate speech among peers. To pursue these goals, HateLess aims to enhance professional competences (e.g., factual knowledge about hate speech), self-competences (e.g., counter-speech, self-efficacy, coping strategies), emotional competences (e.g., empathy, moral engagement), social competences (e.g., cooperative competencies), and methodological competences (e.g., ethical media competences). HateLess can be considered a multilevel program that combines activities on the individual level (e.g., developing skills adolescents need to deal with hate speech), classroom level (e.g., establishing anti-hate speech class rules and norms), school level (e.g., school-wide student activities to raise awareness about hate speech among students and teachers), and community level (e.g., presentation by students during parents’ evening).

HateLess was developed by recognizing a variety of theoretical backgrounds that deal with the personal development of humans, such as the Socio-ecological Theory (Bronfenbrenner, 1979), the Theory of Planned Behavior (Ajzen, 1991), the Theory of Moral Disengagement (Bandura et al., 1996), and the Socio-cognitive Learning Theory (Bandura, (1977)). For example, based on core ideas of the Socio-cognitive Learning Theory (Bandura, (1977)), the five HateLess Heroes, Anura, Bennet, Carla, Hamza, and Laura, who are between 13 and 14 years old and frequently appear throughout the HateLess materials were created. These protagonists guide the students through the program, enhance their motivation, and offer role models. The protagonists have different interests, hobbies, and personality traits and come from family and social backgrounds with diverse origins, ethnicities, and religions to enhance the participants’ likelihood of identifying with them. Some characters or their relatives are affected by hate speech and experience social disadvantages or discrimination. Thus, it makes the topic more accessible for the students, strengthens their empathy, and increases its relevance to adolescents’ everyday lives.

In addition to the theoretical backbone, empirical findings concerning hate speech among adolescents (e.g., Ballaschk et al., 2021; 2022; Krause et al., 2021; Wachs, Wettstein et al., 2022), previous findings and recommendations from prevention research and results about evaluated prevention programs from related fields, such as bullying and discrimination, were reviewed, and effective strategies were synthesized (e.g., Bradshaw, 2015; Domínguez-Martínez & Robles, 2019; Gaffney et al., 2019).

### HateLess Program Components and Didactical Material

HateLess consists of five modules, each planned for one school day, containing three components, which last 90 minutes each. The five successive modules are captioned with a guiding question:

#### Module 1: What is Hate Speech?

The first module starts with implementing classroom rules to ensure a positive learning environment during the project week where all students feel mutually respected, comfortable, and safe. The classroom rules are developed by all students and are made visible as posters in the classroom to serve as reminders. Afterward, the module leads to an in-depth understanding of the phenomenon of hate speech by, for example, discussing the main characteristics of hate speech, different forms of hate speech (e.g., homophobic, xenophobic hate speech), and learning the differences between hate speech and the right to freedom of opinion and expression.

#### Module 2: Why does Hate Speech Exist?

This module engages with potential causes and motives of hate speech perpetration while also considering the relevance of the social norms students encounter in their social environment (e.g., classroom). The students also reflect and analyze group affiliations and characteristics regarding their perceptions of normality, being different, and the social construction of normality, thus uncovering discrimination and privileges and reflecting on these. The students learn to adopt respectful attitudes toward differences through direct engagement with diversity. Furthermore, students train their reflective competencies by critically questioning existing perceptions of normality. In the second module, the students also grapple with digital mechanisms (e.g., fake news, toxic online disinhibition, and echo chambers) that have the potential to amplify hate speech.

#### Module 3: Which Consequences can Hate Speech Have?

In the third module, the students learn about the possible consequences of hate speech for individuals, their class community, and society. By considering five specific everyday areas (e.g., social media, gaming, school, movies, and music), the students reflect on the impact of hate speech in their daily life. With the help of a short movie that presents a case of hate speech in schools, the participants’ understanding of how people feel who become targets of hate speech should be enhanced, hence increasing their ability for empathy and establishing anti-hate speech norms. After that, the students work on various constructive coping strategies to deal with hate speech victimization and reflect on the necessary internal and external resources to facilitate effective coping and learn about ways of reducing the consequences of hate speech.

#### Module 4: How can We Deal with Hate Speech?

During the fourth module, civic courage is introduced, and the students explore how to intervene in hate speech effectively and support classmates who become targets of hate speech, thus reducing passive bystanding, enhancing counter-speech, and increasing the responsibility of each student to create a respectful and inclusive classroom environment. In addition, the students learn about constructive ways of arguing and the basics of non-violent communication, thus enhancing their skills in effective forms of arguing without using hate speech. Also, students learn how to understand their feelings and express critics without hurting the feeling of others.

#### Module 5: How do We Become a HateLess School?

On the last day of the program, the students do small projects and activities on a school level (e.g., designing flyers, planning an exhibition, initiating a student society, producing a podcast episode, or a short video for the school website). In this way, the students share their recently acquired knowledge and expertise with students in different classes and grades, with their parents or guardians, and become peer educators within their school. The last day of the project can lay the foundation for long-term preventative measures on a school level.

HateLess includes a variety of methods, such as discussion, presentations, videos, presenting posters, and exercises done in dyads and small groups by using collaborative and cooperative learning methods. The didactical core of HateLess is a modularized manual (Krause et al., 2022) that helps teachers implement the program independently at their schools. The manual includes segments with background information regarding hate speech, detailed lesson plans, and various materials that facilitate the implementation of the program at school and helps to reduce the necessary preparation time for teachers. Each HateLess component contains a description of a specific learning goal divided into different subordinate goals and a short didactic commentary, which summarizes the lesson plan, gives further advice for the implementation, and proposes differentiation possibilities. Furthermore, various multimedia materials were developed for the HateLess program (e.g., several animated videos, PowerPoint slides). All materials are accessible as open educational resources via a website (www.hateless.de).

### Increasing Empathy, Self-efficacy, and Counter-speech through HateLess

#### Empathy

Empathy is the ability to understand and feel the cognitive and affective experiences of others (Batson, 2009). Evidence from bullying prevention research shows that interventions can effectively increase empathy. For example, the Finnish bullying prevention program, KiVa, successfully increased (affective) empathy after five months of implementation (Saarento et al., 2015). Similarly, the anti-cyberbullying prevention program, Media Heroes, showed empathy-raising effects on empathy for victims of cyberbullying among high school students from Germany (Schultze-Krumbholz et al., 2016).

Empathy should be fostered through HateLess, by dealing with the consequences of hate speech victimization, for example, by watching a short film or conducting role-plays, comprehending the victim’s experience, discussing how the targets feel, and learning about the negative impact of hate speech victimization on the victim’s well-being. In addition, various short stories will be read and discussed in groups to increase participants’ ability to understand and share the emotions of minorized peers targeted by hate speech. Another strategy to increase empathy is “One step forward.” In this method, the teacher hands out role cards with portraits of different people representing a wide range of diversity and a short description of their social background. The teacher then reads out different theses (e.g., “Can you publicly hold hands with the person you are in love with?”), which the participants should either agree or disagree with based on their role. They make their votes clear by moving forward one step if they agree or by standing still. This method triggers an understanding of inequality, discussions in the classroom about how different people experience discrimination regularly, how societies construct *normality*, and whether the assuming character might be exposed to a higher risk of experiencing hate speech.

#### Self-efficacy

Self-efficacy can be defined as one’s perception of one’s ability to perform a particular behavior (Bandura, 1982). There is some evidence that bullying prevention programs can enhance young people’s self-efficacy toward intervening in bullying in favor of the victim. For example, the KiVA program showed to be effective in increasing young people’s self-efficacy for defending victims of bullying after nine months of implementation (Kärnä et al., 2011), and a curriculum‐based anti‐bullying intervention program among Greek students revealed empirical evidence that young people’s self-efficacy can be increased through school-based interventions (Andreou et al., 2007).

An example of how HateLess might increase self-efficacy is by using role-play to allow adolescents to explore various strategies to intervene in hate speech, understand how the social environment reacts to it, and discuss the potential effects of each strategy, including potential hurdles, benefits, and challenges. Self-efficacy is also addressed as part of the exercise “Resources Cards.” Participants’ awareness of their personal, social, material, and socio-spatial resources should be increased through this exercise. At the same time, adolescents discuss what a person targeted by hate speech would need as support and which support systems are available within the classroom, at the school, and in the external social environment. Self-efficacy is also supported by the “Warm Shower” method used in HateLess. In this method, the learning group sits in a circle of chairs, with one in the center of the circle. Everyone, in turn, sits down on this chair and receives a “Warm Shower” from the others in the form of a compliment on one or more positive characteristics.

#### Countering Hate Speech

Countering hate speech (counter-speech) is defined as a form of citizen-based response to hateful content to discourage it, stop it, or provide support for the victim by, for example, pointing out logical flaws in the hateful comment or using facts to counteract misinformation (Garland et al., 2022). Previous research revealed that defending behavior in favor of the victim can be directly increased through school-based interventions (Saarento et al., 2015; Zambuto et al., 2020).

HateLess aims at increasing counter-speech through various methods and learning occasions. For example, counter-speech is addressed by introducing the concept of civic courage, increasing participants’ feeling of responsibility to counteract hate speech in the classroom, and reducing passive bystanding. Short stories dealing with hate speech incidents might also increase adolescents’ ability to counter hate speech. In group work, the participants are asked to answer questions about the passive bystanders in the stories, develop ideas about constructive reactions, and discuss which behaviors might be appropriate and helpful in addressing the described hate speech incidents. In addition, HateLess includes training on non-violent communication, including a reflection on identifying feelings and exercises on expressing needs without hurting others or formulating criticism without hurting others. The participants also learn to assess when counter-speech is advised, for example, by discussing fictitious online comments to determine whether they are hate speech or expressions of opinion, thus learning the differences between critical but legitimate statements and hate speech.

### The Role of Empathy and Self-Efficacy in Countering Hate Speech

Often interventions do not alter the targeted outcomes directly but indirectly through mediating variables (Saarento et al., 2015), which might also be the case in the present study. It is expected that HateLess does not only directly affect adolescents’ counter-speech but also indirectly influences counter-speech via empathy and self-efficacy.

It can be assumed, for example, that understanding and feeling the affective situation of hate speech victims by vicariously experiencing their emotional distress might favor benign feelings for the victims and thus increase counter-speech. This assumption is in line with burgeoning research on hate speech that revealed that higher levels of empathy were negatively associated with the perpetration and acceptance of online hate speech (Celuch et al., 2022; Wachs, Bilz, et al., 2022). Another study revealed that adolescents who can understand and share the emotions of peers who become targets of hate speech were more likely to counter hate speech in favor of the victim (Wachs et al., 2023). These initial findings of hate speech research align with numerous studies from related fields, which found unequivocally that higher levels of empathy were positively related to defending victims of bullying against peers who perpetrate bullying (Álvarez-García et al., 2021; Gönültaş & Mulvey, 2022; Shen et al., 2022). This assumption is also supported by previous empirical work that showed that empathy was positively associated with prosocial behavior and negatively linked to intolerance and prejudice (Boag & Carnelley, 2016; Pettigrew & Tropp, 2008). Empirical research investigating the indirect effects of participating in an intervention on defending behavior via empathy is scarce. However, one study found that adolescents’ willingness to intervene in bullying through participation in a virtual enhanced anti-bullying prevention program was mediated through increases in empathy (Ingram et al., 2019).

Whether adolescents engage in counter-speech might not only be influenced by their compassion for the targeted person but might also be related to the self-perceived ability to intervene efficaciously. Self-efficacy might be necessary for adolescents’ counter-speech, as they are more likely to intervene if they have confidence in their capacity to do so successfully. Indeed, initial research found a positive link between self-efficacy and counter-speech (Wachs et al., 2023). This finding aligns with myriad empirical research positing that adolescents who perceive themselves as self-efficacious in dealing with social conflicts, such as bullying (Sjögren et al., 2020; Thornberg et al., 2020), are more likely to intervene in favor of targeted peers. There is also some indication that the relationship between participating in a role-play intervention and adolescents’ defender intention in favor of victimized peers is mediated by defender self-efficacy (Abbott et al., 2020).

## Current Study

Not much is known yet about effective strategies to empower adolescents to deal proactively with hate speech incidents. To this end, the present study aimed to evaluate the short-term effects of the newly developed anti-hate speech program, HateLess. More specifically, the present study’s first aim was to evaluate the effectiveness of HateLess on adolescents’ self-reported empathy toward victims of hate speech, self-efficacy for intervening in hate speech, and counter-speech. Drawing upon the previous research, it was hypothesized that the reported levels of empathy would increase in the intervention group but not in the control group (Hypothesis 1); and that the reported levels of self-efficacy will increase in the intervention group but not in the control group (Hypothesis 2); and that the reported levels of counter-speech will increase in the intervention group but not in the control group (Hypothesis 3). This study’s second purpose was to understand the underlying mechanisms of the effectiveness in successfully increasing counter-speech through participation in HateLess. Since empathy and self-efficacy are hallmark characteristics of adolescents’ prosocial behavior and initial research indicates a positive link, a multilevel mediation model was used to test the hypothesis that being in the intervention group will positively predict higher levels of counter-speech via empathy and self-efficacy (Hypothesis 4).

## Method

### Participants

A priori power analysis with G*Power (Faul et al., 2007) revealed that a sample consisting of at least 782 participants (α = 0.05, Power = 0.80) was required to detect small to medium effects. The sample included 820 adolescents between 12 and 16 years old (*M* = 13.27, *SD* = 1.04) in grades 7 to 9 (7th grade: 39.8%; 8th grade: 40.5%, 9th grade: 19.8%) from 37 classes in 11 schools across Germany. Regarding gender, 47.3% self-identified as female, 51% as male, 1.2% as gender diverse, and 0.5% did not indicate their gender. Overall, 35.6% had an immigrant background, 63.3% did not have an immigrant background, and 1.1% did not indicate whether they had an immigrant background. The intervention group consisted of 567 participants (*M*_age_ = 13.24, *SD* = 1.07, 45.9% self-identified as female, 35.8% had an immigrant background) and the control group of 253 participants (*M*_age_ = 13.35, *SD* = 0.97, 50.6% self-identified as female, 35.2% had an immigrant background). The intervention and control group did not significantly differ regarding age, gender, or immigrant background.

### Measures

#### Empathy for Victims of Hate Speech

The instrument for measuring empathy was adapted from Knauf et al. (2018). Originally, this instrument referred to bullying in the introduction. In the revised version used in the present study, hate speech was referred to in the introduction of the instrument: “When I see classmates being insulted or attacked by other classmates because of their skin color, origin, religion, sexual orientation, or gender….” For empathy, six items were included to reflect different aspects of empathy, including perspective-taking (e.g., “I realize how badly they are doing”), affective empathy (e.g., “It hurts me, too”), and empathic concern (e.g., “It makes me want to comfort them”). Items were rated on a five-point scale from “absolutely disagree” (1) to “absolutely agree” (5). The internal consistency of the empathy scale in the current sample was Cronbach’s alphas _pretest_ = 0.89 / McDonald’s Omegas _pretest_ = 0.89 and Cronbach’s alphas _posttest_ = 0.90 / McDonald’s Omegas _posttest_ = 0.91. CFAs confirmed a good fit: CFA _pretest_: χ^2^ = 46.04, *df* = 5, *p* < 0.001, CFI = 0.96, TLI = 0.93, RMSEA = 0.06 [0.04, 0.10], SRMR = 0.03. CFA _posttest_: χ^2^ = 43.04, *df* = 5, *p* < 0.001, CFI = 0.97, TLI = 0.95, RMSEA = 0.07 [0.03, 0.12], SRMR = 0.02.

#### Self-efficacy Toward Intervening in Hate Speech

A scale developed by Knauf et al. (2018) was adapted to measure adolescents’ self-efficacy toward intervening in hate speech. As for the empathy scale, hate speech was referred to in the introduction. Three items were used: “I can immediately think of ways to do something against it,” “I have ideas about how I can help them,” and “I know that there are things I can do to improve the situation.” Responses could be rated on a five-point scale from “absolutely disagree” (1) to “absolutely agree” (5). The internal consistency of the self-efficacy scale in the current sample was Cronbach’s alphas _pretest_ = 0.85 / McDonald’s Omegas _pretest_ = 0.85 and Cronbach’s alphas _posttest_ = 0.87 / McDonald’s Omegas _posttest_ = 0.88. CFAs confirmed a good fit: CFA _pretest_: χ^2^ = 6.81, *df* = 2, CFI = 0.98, TLI = 0.98, RMSEA = 0.06 [0.02, 0.12], SRMR = 0.07. CFA _posttest_: χ^2^ = 5.01, *df* = 2, CFI = 0.99, TLI = 0.99, RMSEA = 0.04 [0.01, 0.07], SRMR = 0.03.

#### Counter-speech

A scale developed by Wachs et al. (2023) was used to measure counter-speech (countering hate speech). Participants rated the following four items: “I tell the person that such statements are hurtful, “I ask the person to stop”; “I try to get the person to think by asking specific questions”; and “I say that the person is spreading false information (fake news).” All items could be answered on a five-point response scale from “strongly disagree” (1) to “strongly agree” (5). The internal consistency of the counter-speech scale in the current sample was Cronbach’s alphas _pretest_ = 0.84 / McDonald’s Omegas _pretest_ = 0.84 and Cronbach’s alphas _posttest_ = 0.86 / McDonald’s Omegas _posttest_ = 0.85. CFAs confirmed a good fit: CFA _pretest_: χ^2^ = 10.64, *df* = 2, CFI = 0.97, TLI = 0.93, RMSEA = 0.06 [0.02, 0.12], SRMR = 0.03. CFA _posttest_: χ^2^ = 7.84, *df* = 2, CFI = 0.99, TLI = 0.97, RMSEA = 0.07 [0.03, 0.13], SRMR = 0.02.

#### Demographic Variables

Participants were asked to identify their age and gender (boy, girl, gender diverse). The immigrant background was assessed by asking whether the participant or one of their parents was born in a country other than Germany. Participants were classified as having an immigrant background if at least one parent had been born outside Germany, which follows the official German definition of immigrant background (Statistisches Bundesamt Destatis, 2022).

### Procedure

**Fig. 1:**
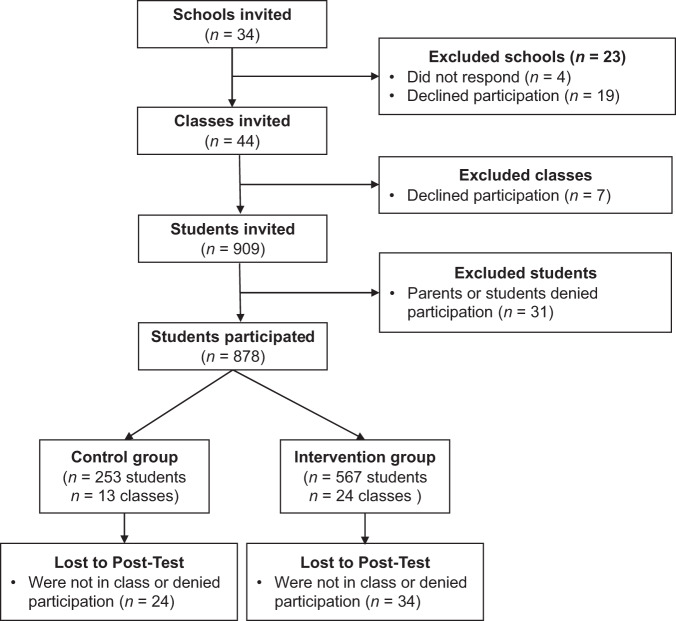
Flowchart of the Recruitment and Allocation

Ethical approval for this study was obtained from the data protection officer and the educational authority of the Federal States of Berlin and Brandenburg, Germany. Overall, 34 schools were contacted via phone and e-mail, indicating that they had been randomly selected to participate in the study. Four of those 34 schools did not respond, and 19 declined participation, citing a lack of resources, high regional COVID-19 infection rates at the time of contact, or commitment to other prevention programs. Participation at the school level was 32% (*N* = 11). Schools were selected based on the staff’s willingness to accommodate scheduling and confirmation that no other anti-hate speech or anti-bullying prevention programming was implemented in the participating classes. In total, 44 classes were invited to participate in the intervention study. Seven classes declined participation, leading to a participation rate at the classroom level of 84% (*N* = 37). Overall, 909 students were invited to participate in this study, from which 31 refused to participate, leading to a response rate of 96% (*N* = 878) at the student level. Of these 878 students who participated in the pretest (T1), 58 did not participate in the posttest (T2), leading to a sample size of 820 students who took part in the pretest and posttest evaluation. Figure 1 shows the rates of participation at each step.

To increase the willingness to participate in this intervention study, schools were offered the possibility to assign their participating classes to the intervention conditions randomly. All schools were provided with teaching materials and access to multimedia content. Data were collected between June and November 2022 via the computer-assisted personal interview technique during a regular school lesson by trained research assistants. Pretest and posttest data were collected in each class either before summer break or after to ensure that the classroom composition did not change during the intervention study. Students in German high schools usually remain with the same classmates throughout the school day and over several school years. Data for this intervention study were collected one week before the intervention (pretest; T1) and one month after (posttest; T2).

### Data Analysis

The missing data of each variable (i.e., empathy, self-efficacy, and counter-speech) were examined using SPSS 29.0 (IBM, 2022). Missing data ranged from 0.2% (T1 empathy) to 4.9% (T2 self-efficacy). According to the Little’s Missing Completely at Random (MCAR) test, data were not likely to be MCAR, *χ*^*2*^ = 192.32, *df* = 40, *p* < 0.001. Hence, the multiple imputation procedure in SPSS 29.0 (IBM, 2022) was used to impute missing values by sequential regression imputation based on five data sets.

Before testing our hypotheses, descriptive statistics, bivariate correlations, and independent *t*-tests were computed to investigate the study’s variables and differences in means between the pretest (T1) and posttest (T2). To test whether HateLess was successful in increasing empathy (H1), self-efficacy (H2), and counter-speech (H3) from pretest to posttest three separate 2 (pretest vs. posttest) × 2 (intervention group vs. control group) repeated measures ANOVAs were performed with time (pretest vs. posttest) as the within-subjects variable and group as the between-subjects variable. The interpretation of the interaction terms in the repeated measures ANOVA was used to evaluate the program’s effectiveness. The partial eta-square (η_p_^2^) was used as an indicator of the size of the effect, according to which a η_p_^2^ ≤ 0.05 can be considered a ‘small’ effect size, 0.06 ≤ η_p_^2^ ≤ 0.14 represents a ‘medium’ effect size, and η_p_^2^ > 0.14 is a ‘large’ effect size (Cohen, 1988).

H4, namely, whether the effect of being a member of the intervention group on counter-speech was partially mediated by empathy and self-efficacy, was tested in two stages using Mplus 8.7 (Muthén & Muthén, 2021). First, a model with a random intercept only was used to estimate the intraclass correlation coefficients (ICC). Second, a multilevel model that included group assignment (intervention vs. control group) as the independent variable, T2 empathy and T2 self-efficacy as mediating variables, and T2 counter-speech as the outcome while controlling T2 empathy, T2 self-efficacy, and T2 counter-speech for variation in T1 empathy, T1 self-efficacy, T1 counter-speech, age, gender, and immigrant background at the student level (L1) was estimated. No variables were included at classroom level (L2).

A multilevel CFA was estimated to confirm the measurement model’s adequacy. The goodness-of-fit was examined by considering the following indices: The Comparative Fit Index (CFI), the Tucker-Lewis index (TLI), the Root Mean Square Error of Approximation (RMSEA), and the Standardized Root Mean Square Residual (SRMR). The quality of each model was evaluated using typical cut-off scores reflecting good and adequate fit of the data, respectively: CFI and TLI > 0.95 and 0.90; RMSEA < 0.06 and 0.08, and SRMR < 0.10 and 0.05 (Hu & Bentler, 1999).

## Results

### Preliminary Analyses

To examine possible baseline differences between the experimental conditions (intervention group vs. control group), *t*-tests were conducted separately for each outcome at the pretest. Results showed no significant differences across the two experimental conditions for empathy, *t*(818) = 0.812, *p* = 0.417; self-efficacy, *t*(818) = 1.60, *p* = 0.109; and counter-speech, *t*(818) = 0.055, *p* = 0.956, confirming the comparability of the groups across the conditions. Table 1 shows the correlations among the study’s main variables. All correlations were in the expected direction.

**Table 1:** Pearson’s Bivariate Correlations, Descriptive Statistics, and ICC

Variable	1	2	3	4	5	6
Empathy (T1)	–	0.52^**^	0.53^**^	0.39^**^	0.55^**^	0.44^**^
Empathy (T2)		–	0.33^**^	0.52^**^	0.40^**^	0.52^**^
Self-efficacy (T1)			–	0.45^**^	0.55^**^	0.37^**^
Self-efficacy (T2)				–	0.34^**^	0.51^**^
Counter-speech (T1)					–	0.57^**^
Counter-speech (T2)						–
M	2.63	2.80	2.57	2.85	2.71	2.93
SD	0.77	0.78	0.79	0.81	1.04	1.09
ICC	0.09	0.13	0.06	0.09	0.07	0.09

^**^p < 0.001. *N* = 820. T1 = Pretest, T2 = Posttest

### Effectiveness of HateLess in Increasing Empathy, Self-efficacy, and Counter-speech

**Fig. 2:**
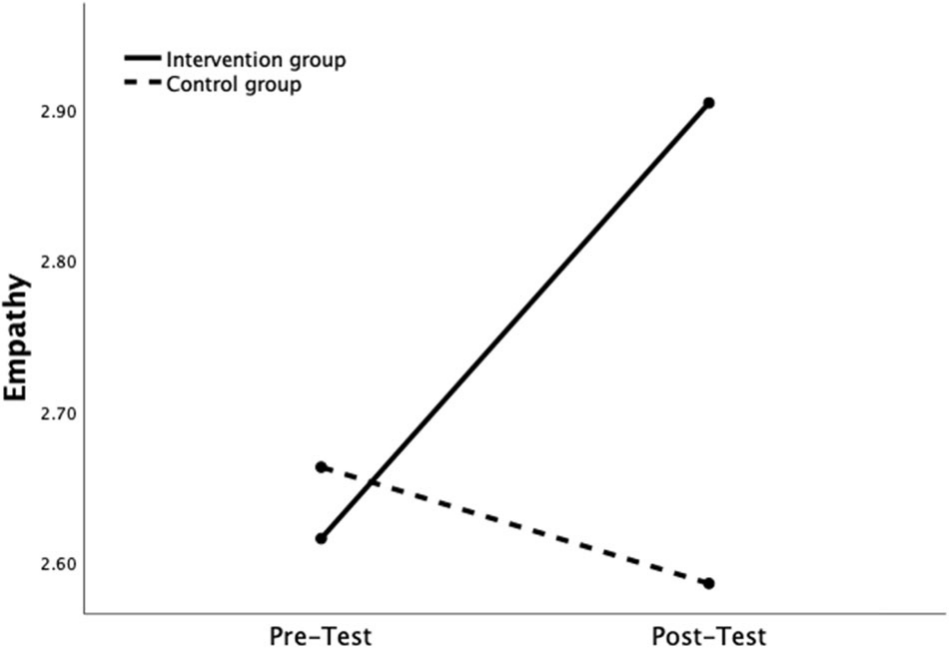
Empathy Change over time as a Function of Group Assignment

The present study’s first aim was to investigate the short-term success of the prevention program HateLess on adolescents’ self-reported empathy, self-efficacy, and counter-speech. The first repeated measures ANOVA had empathy as the outcome. Results showed a significant main effect of time, *F*(1,818) = 14.05, *p* < 0.001, with a small effect size, η_p_^2^ = 0.02, and a significant main effect of group, *F*(1, 818) = 7.08, *p* = 0.008, with a small effect size, η_p_^2^ = 0.01. In addition, a significant time × group interaction effect was found, *F*(1, 818) = 41.97, *p* < 0.001, with a small effect size, η_p_^2^ = 0.05. A simple effects analysis was used to probe this interaction. Results showed a significant change over time for those in the intervention group, *F*(1, 818) = 84.74, *p* < 0.001, with medium effect size, η_p_^2^ = 0.09, but not for those in the control group, *F*(1, 818) = 2.69, *p* = 0.101, η_p_^2^ = 0.00. Examination of the means revealed a significant increase in empathy (∆*M* = 0.29, *p* < 0.001) between pretest (*M* = 2.61, *SE* = 0.03, 95% CI [2.55, 2.68]) and posttest (*M* = 2.90, *SE* = 0.03, 95% CI [2.84, 2.97]) in the intervention group and a nonsignificant decrease of empathy (∆*M* = −0.08, *p* = 0.101) between pretest (*M* = 2.66, *SE* = 0.05, 95% CI [2.56, 2.75]) and posttest (*M* = 2.58, *SE* = 0.05, 95% CI [2.49, 2.65], see Fig. 2) in the control group. In conclusion, participation in HateLess was successful in the short term at increasing empathy, supporting our Hypothesis 1.

**Fig. 3:**
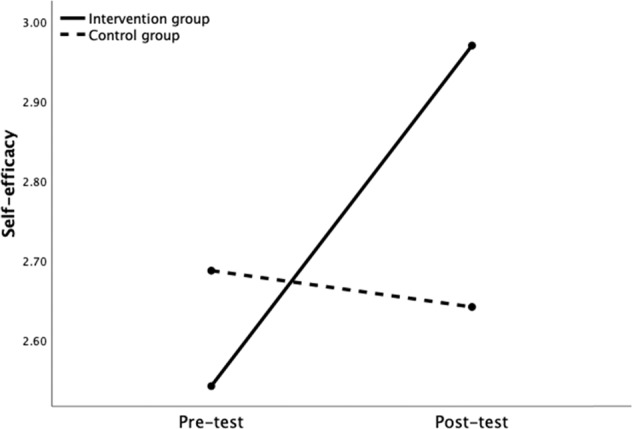
Self-efficacy Change over time as a Function of Group Assignment

The second repeated measures ANOVA had self-efficacy as the outcome. Results showed a significant main effect of time, *F*(1, 818) = 41.25, *p* < 0.001, with a small effect size, η_p_^2^ = 0.05, and a significant main effect of group, *F*(1, 818) = 5.67, *p* = 0.017, with a small effect size, η_p_^2^ = 0.01. In addition, a significant time × group interaction effect was found, *F*(1, 818) = 49.40, *p* < 0.001, with medium effect size, η_p_^2^ = 0.07. A simple effects analysis was used to probe this interaction. Results showed a significant change over time for those in the intervention group, *F*(1, 818) = 146.61, *p* < 0.001, with a large effect size, η_p_^2^ = 0.15, but not for those in the control group, *F*(1, 818) = 0.133, *p* = 0.716, η_p_^2^ = 0.00. Examination of the means revealed a significant increase in self-efficacy (∆*M* = 0.43, *p* < 0.001) between pretest (*M* = 2.54, *SE* = 0.03, 95% CI [2.48, 2.61]) and posttest (*M* = 2.97, *SE* = 0.03, 95% CI [2.90, 3.03]) and a nonsignificant decrease of self-efficacy (∆*M* = −0.04, *p* = 0.395) between pretest (*M* = 2.68, *SE* = 0.05, 95% CI [2.58, 2.79]) and posttest (*M* = 2.64, *SE* = 0.05, 95% CI [2.54, 2.74], see Fig. 3) in the control group. In conclusion, participation in HateLess was successful in the short term at increasing self-efficacy, supporting our Hypothesis 2.

**Fig. 4:**
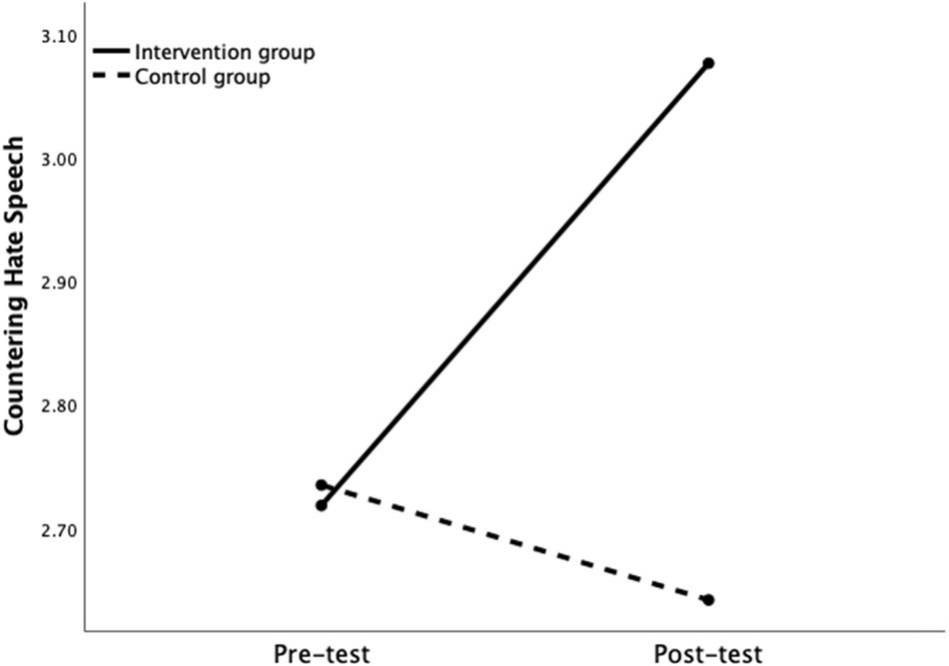
Countering Hate Speech Change over time as a Function of Group Assignment

The third repeated measures ANOVA had counter-speech as the outcome. Results showed a significant main effect of time, *F*(1, 818) = 14.33, *p* < 0.001, with a small effect size, η_p_^2^ = 0.02, and a significant main effect of group, *F*(1, 818) = 9.13, *p* = 0.003, with a small effect size, η_p_^2^ = 0.01. In addition, a significant time×group interaction effect was found, *F*(1, 818) = 35.57, *p* < 0.001, with a small effect size, η_p_^2^ = 0.05. A simple effects analysis was used to probe this interaction. Results showed a significant change over time for those in the intervention group, *F*(1, 818) = 77.03, *p* < 0.001, with a medium effect size, η_p_^2^ = 0.09, but not for those in the control group, *F*(1, 818) = 1.72, *p* = 0.191, η_p_^2^ = 0.00. Examination of the means revealed a significant increase in counter-speech (∆*M* = 0.36, *p* < 0.001) between pretest (*M* = 2.72, *SE* = 0.04, 95% CI [2.63, 2.81]) and posttest (*M* = 3.08, *SE* = 0.04, 95% CI [2.98, 3.17]) in the intervention group and a nonsignificant decrease of counter-speech (∆*M* = −0.09, *p* = 0.140) between pretest (*M* = 2.73, *SE* = 0.07, 95% CI [2.60, 2.86]) and posttest (*M* = 2.64, *SE* = 0.07, 95% CI [2.51, 2.78], see Fig. 4) in the control group. In conclusion, participation in HateLess was successful in the short term at increasing counter-speech, supporting our Hypothesis 3.

### Effects of HateLess on Counter-speech via Empathy and Self-efficacy

The previous analyses showed that HateLess successfully increased empathy, self-efficacy, and counter-speech among adolescents from the pretest to the posttest. In the next step, a multilevel mediation model for understanding better how HateLess was successful in increasing counter-speech was estimated. A model with a random intercept only was used to estimate the ICC. This model showed that the ICC for empathy (T2) was 0.13, the ICC for self-efficacy (T2) was 0.09, and the ICC for counter-speech (T2) was 0.09. To confirm the adequacy of the measurement model, a multilevel confirmatory factor analysis, including T2 empathy, T2 self-efficacy, and T2 counter-speech, was conducted. The CFA supported the adequacy of the measurement model, χ^2^ = 192.16, *df* = 121, *p* < 0.001, CFI = 0.96, TLI = 0.96, RMSEA = 0.032, SRMR = 0.066. In the next step, the mediation model was tested. The proposed multilevel mediation model explained 44% of the total variance in counter-speech (*R*^2^ = 0.441), 31% of the total variance in empathy (*R*^2^ = 0.312), and 27% of the total variance in self-efficacy (*R*^2^ = 0.272). The results showed that being in the intervention group predicted higher T2 levels of empathy (*B* = 0.34, *SE* = 0.06, *p* < 0.001), higher T2 levels of self-efficacy (*B* = 0.38, *SE* = 0.07, *p* < 0.001), and higher T2 levels of counter-speech (*B* = 0.20, *SE* = 0.07, *p* = 0.002) compared to the control group while controlling for T1 levels of empathy, self-efficacy, and counter-speech. In addition, T2 levels of empathy (*B* = 0.31, *SE* = 0.06, *p* < 0.001) and T2 levels of self-efficacy (*B* = 0.32, *SE* = 0.05, *p* < 0.001) were positively associated with T2 levels of counter-speech. The indirect effects of being in the intervention group on T2 counter-speech through T2 empathy (*B* = 0.10, *SE* = 0.03, 95% CI [0.05, 0.15]) and T2 self-efficacy (*B* = 0.12, *SE* = 0.03, 95% CI [0.07, 0.19]) were significant. That is, being a member of the intervention group predicted increases in T2 empathy and self-efficacy, which in turn was positively associated with adolescents’ T2 counter-speech while controlling for T1 levels. In conclusion, these results confirmed that HateLess successfully changed counter-speech directly and indirectly through increased empathy and self-efficacy, supporting our Hypothesis 4. Table 2 shows the full results of the mediation model, including all effects of the control variables on the study’s main variables.

**Table 2:** Results of the Multilevel Mediation Model

Predictor	Mediator	Outcome	B (SE)	*p*
*Direct Effects*				
Group assignment ^Intervention^	Empathy (T2)		0.34 (0.06)	<0.001
Group assignment ^Intervention^	Self-efficacy (T2)		0.38 (0.07)	<0.001
Group assignment ^Intervention^		Counter-speech (T2)	0.20 (0.07)	0.002
	Empathy (T2)	Counter-speech (T2)	0.31 (0.06)	<0.001
	Self-efficacy (T2)	Counter-speech (T2)	0.32 (0.05)	<0.001
*Indirect Effects*				
Group assignment ^Intervention^	Empathy (T2)	Counter-speech (T2)	0.10 (0.03) 95% CI [0.05, 0.15]	<0.001
Group assignment ^Intervention^	Self-efficacy (T2)	Counter-speech (T2)	0.12 (0.03) 95% CI [0.07, 0.19]	<0.001
*Control variables*				
Age		Counter-speech (T2)	−0.04 (0.03)	0.150
Age		Empathy (T2)	−0.01 (0.02)	0.791
Age		Self-efficacy (T2)	−0.04 (0.03)	0.128
Gender ^Girls^		Counter-speech (T2)	0.27 (0.07)	<0.001
Gender ^Girls^		Empathy (T2)	0.23 (0.03)	0.008
Gender ^Girls^		Self-efficacy (T2)	−0.09 (0.07)	0.213
Immigrant background ^Yes^		Counter-speech (T2)	−0.03 (0.05)	0.587
Immigrant background ^Yes^		Empathy (T2)	−0.03 (0.04)	0.412
Immigrant background ^Yes^		Self-efficacy (T2)	0.03 (0.04)	0.472
Counter-speech (T1)		Counter-speech (T2)	0.43 (0.04)	<0.001
Empathy (T1)		Empathy (T2)	0.53 (0.04)	<0.001
Self-efficacy (T1)		Self-efficacy (T2)	0.48 (0.04)	<0.001

T1 = Pretest, T2 = Posttest. Reference category: group assignment = control group, gender = boys, immigrant background = no

## Discussion

Adolescence is a crucial life stage in developing political identity, civic courage, and democratic values and skills. At the same time, adolescents spend a great deal of time in school. In recent years, diversity has become more visible in schools. This new visibility also has brought up old resentments and further backlashes. As central societal institutions, schools can prepare young people to adapt to an evolving world and embrace diversity. To meet this task, however, schools need effective strategies. This is where the present study comes in by evaluating the effectiveness of HateLess, the first theoretically and empirically grounded program targeting hate speech among adolescents. More specifically, the present study a) evaluated the effects of HateLess on adolescents’ empathy toward victims of hate speech, their self-efficacy toward intervening in hate speech and counter-speech, and b) analyzed the effects of participation in HateLess on counter-speech via empathy and self-efficacy to understand the underlying mechanism of HateLess’s effectiveness in increasing counter-speech.

Regarding the first aim, the intervention data clearly showed that adolescents who received the HateLess intervention reported higher levels of empathy for victims of hate speech, felt more self-efficacious toward intervening and countered more often hate speech, compared with adolescents in the control group. These positive intervention effects, which support Hypotheses 1 to 3, are the first empirical evidence for the effectiveness of HateLess in empowering adolescents to deal with hate speech in schools. The effects were small for empathy and counter-speech and moderate for self-efficacy, which is consistent with previous research on school-based anti-bullying interventions that showed similar effects on empathy (Saarento et al., 2015; Schultze-Krumbholz et al., 2016), self-efficacy (Andreou et al., 2007; Kärnä et al., 2011), and defending of victims (Ingram et al., 2019; Zambuto et al., 2020).

Turning to the second aim, the present study sought to understand the underlying mechanisms of the effectiveness in successfully increasing counter-speech through participation in HateLess. The main variables in this path model were controlled for baseline levels of empathy (T1), self-efficacy (T1), and counter-speech (T1), age, gender, and immigrant background, to add additional credibility to the results. The investigation of mechanisms of change confirmed Hypothesis 4 and indicated that manipulating empathy and self-efficacy through HateLess can positively influence counter-speech. The findings highlight the need to provide adolescents with the ability to respond emotionally and understand the emotions of victims of hate speech to increase adolescents’ readiness to argue, disagree or express an opposing view to hateful statements or content. The present result is supported by initial research showing a negative association between empathy and hate speech perpetration (Celuch et al., 2022; Wachs, Bilz, et al., 2022) and a positive link between empathy and counter-speech (Wachs et al., 2023). Overall, the findings are also mirrored by research that found a negative relationship between empathy and intolerance and prejudice and a positive link between empathy and prosocial behavior (Boag & Carnelley, 2016; Pettigrew & Tropp, 2008). Another finding related to H4 was that adolescents’ belief in their capability to deal with hate speech is positively associated with their engagement against hate speech. This finding is in accordance with a rich body of research on bullying, highlighting the crucial role of self-efficacy in actual defending behavior in favor of targets of bullying (Sjögren et al., 2020; Thornberg et al., 2020) and initial research on the relationship between self-efficacy and counter-speech (Wachs et al., 2023).

Finally, the mediation analysis showed that HateLess effectively increased counter-speech by targeting empathy and self-efficacy. Thus, empathy and self-efficacy might be relevant factors to consider that transmit the effects of HateLess on counter-speech. These findings highlight the need to focus on the outcome of interest and target proximal factors related to the specific outcome. The results regarding the indirect effects are similar to previous bullying research by Ingram et al. (2019), who also found evidence for an indirect effect between participating in a prevention program and defending behavior via empathy. Likewise, the findings align with other bullying research that revealed that self-efficacy mediated the relationship between participating in a role-play intervention and defender intentions (Abbott et al., 2020). Adding to this line of research, the present study revealed that participation in HateLess did not have only a direct positive effect on counter-speech but also an indirect effect via empathy and self-efficacy.

### Practical Implications for Anti-hate Speech Prevention Programs

Several practical implications for anti-hate speech prevention programs can be drawn from the current study. First, this study suggests that empathy for victims of hate speech, self-efficacy toward intervening in hate speech, and counter-speech are malleable. That is, they can be changed via targeted anti-hate speech intervention programs. Approaches to prevent prejudicial attitudes and intergroup relationships can be distinguished into three groups: Intergroup contact interventions (providing direct contact via, e.g., youth exchange programs or indirect contact via, e.g., reading stories about social out-group members), knowledge-based intervention (e.g., providing information about minorities, and democratic values and principles), and individual skill acquisition (e.g., empathy training) (Beelmann & Lutterbach, 2020). Combining all three approaches, as done in HateLess, might be fruitful in empowering adolescents to deal with hate speech.

Second, it is noteworthy that the found effects were achieved by a classroom-based intervention, independently implemented by teachers and not by trained external experts, albeit coordinated and supported by the research team (e.g., providing copies of worksheets). A benefit of teachers being able to implement HateLess without relying on external trainers is that HateLess allows teachers to bond with their students without focusing solely on teaching the traditional hard skills (e.g., reading, writing). Adolescents who participated in HateLess recognized this as beneficial, as indicated by the responses to whether the teacher-student relationship has positively changed through participation in HateLess. Approximately 32% of participating adolescents reported that their relationship with their teachers had slightly changed positively, and 27% stated that their relationship with their teachers improved significantly. This evaluation study also indicated that schools represent a proper environment to implement the standardized anti-hate speech program HateLess which is essential, as compulsory school attendance allows access to most adolescents in Germany. Being able to implement the program independently also increases the actual implementation because sometimes time-consuming training sessions are not feasible due to limited personal resources. Indeed, lacking personal resources is one of the most often cited barriers schools face when considering prevention programs (World Health Organization, 2019).

Third, regarding feasibility, the findings indicated that cost-efficient anti-hate speech prevention programs could be effective. As there are no training and licensing fees, and all materials can be accessed as open educational resources from a website, the direct costs for conducting HateLess for one class with approximately 30 students are approximately 30$ for printing the manual and worksheets. Also, indirect costs (i.e., school personnel wages) are no additional financial burden to schools as HateLess is implemented during a project week which is regularly conducted once during a school year in German schools.

Finally, the development of HateLess was guided by combining results from diverse research fields (e.g., prejudice, discrimination, antisocial behavior), which showed to be a promising approach because hate speech combines several aspects of these concepts. HateLess might be one step toward developing more holistic prevention programs, often recommended by prevention researchers (e.g., Beelman & Lutterbach, 2020) if follow-up evaluation studies show that HateLess has not only an effect on hate speech but also other forms of antisocial behavior (e.g., bullying, aggressive behavior).

### Limitations and Future Directions

Several limitations of the present study need to be mentioned and addressed in follow-up research. First, the schools conducted the randomization of classes receiving the HateLess intervention. Hence, using this quasi-experimental design does not guarantee a random assignment of the classes. The levels of empathy, self-efficacy, and counter-speech in the intervention and control group at baseline (T1) were compared to address internal validity concerns. The findings revealed no significant differences between the intervention and control group at T1. Nonetheless, follow-up research should try to replicate the present results using a traditional experimental design.

Second, the present study could only investigate short-term effects within one month after the program. These data do not give any information about the stability of these effects over a longer time. Using follow-up data collection is needed to study the long-term impact of HateLess. Third, this study relied on students’ self-report exclusively. Follow-up research might benefit from including peer nominations to investigate, for example, adolescents’ counter-speech. In addition, teacher reports could be used as an additional resource of information on students’ behavioral change through HateLess. Teacher reports could also help clarify implementation problems and the potential effects of the intervention on teachers (e.g., teachers’ self-efficacy toward intervening in hate speech among students). Fourth, while HateLess is effective in increasing empathy, self-efficacy, and counter-speech, the effects were small to moderate, which means that HateLess seems to work for some students but not all. Attention should be devoted to the characteristics of adolescents who did not show changes despite participating in HateLess and identify factors related to HateLess’s intervention failure. Such information can help to develop more tailored and diversified strategies to improve the intervention’s success. Finally, other intrapersonal variables (e.g., involvement in hate speech, coping strategies, social skills, tolerance, attitudes) and contextual variables (e.g., inclusive classroom climate, social norms within classrooms) should be investigated more thoroughly in the future to understand the full impact and potential of HateLess.

## Conclusion

Despite growing attention to hate speech among youth, not much is known about effective strategies to tackle hate speech in schools. The present study addressed this gap in the literature by investigating the effectiveness of the newly developed prevention program HateLess. The results pinpointed the impact of HateLess in increasing adolescents’ empathy, self-efficacy, and counter-speech. There was also some indication that this positive effect of HateLess on empathy and self-efficacy may translate into an increase in counter-speech. However, more research is required to substantiate further the present findings and understand the effectiveness of HateLess. Such research should use a randomized design, a follow-up measure, the effects of HateLess on more intrapersonal (e.g., involvement in hate speech as perpetrator or victim, pro-diversity attitudes), and contextual variables (e.g., inclusive classroom climate), and multiple measures of effectiveness (peer reports, teacher reports, and self-reports). Despite these limitations, the present study’s findings indicate that HateLess is an effective, cost-efficient approach that schools can implement independently and enables them to overcome current challenges and prepare youth for peaceful living in diverse societies.

## Data Availability

The datasets analyzed during the current study are not publicly available but are available from the corresponding author upon reasonable request.
